# Lack of evidence supporting a role of *TMC4*-rs641738 missense variant—*MBOAT7*- intergenic downstream variant*—*in the Susceptibility to Nonalcoholic Fatty Liver Disease

**DOI:** 10.1038/s41598-018-23453-9

**Published:** 2018-03-23

**Authors:** Silvia Sookoian, Diego Flichman, Martin E. Garaycoechea, Carla Gazzi, Julio San Martino, Gustavo O. Castaño, Carlos J. Pirola

**Affiliations:** 1 University of Buenos Aires, Institute of Medical Research A Lanari, Buenos Aires, Argentina; 2 National Scientific and Technical Research Council (CONICET)-University of Buenos Aires. Institute of Medical research (IDIM), Department of Clinical and Molecular Hepatology, Buenos Aires, Argentina; 3 University of Buenos Aires, School of Pharmacy and Biochemistry, Department of Virology, and National Scientific and Technical Research Council (CONICET), Ciudad Autonoma de Buenos Aires, Buenos Aires, Argentina; 4 Hospital de Alta Complejidad en Red El Cruce, Department of Surgery, Buenos Aires, Argentina; 5 University of Buenos Aires, Institute of Medical Research A Lanari, Department of Pathology, Buenos Aires, Argentina; 6 Hospital Diego Thompson, San Martin, Department of Pathology, Buenos Aires, Argentina; 7 Hospital Abel Zubizarreta, Department of Medicine and Surgery, Liver Unit, Buenos Aires, Argentina; 8 National Scientific and Technical Research Council (CONICET)-University of Buenos Aires. Institute of Medical research (IDIM), Department of Molecular Genetics and Biology of Complex Diseases, Buenos Aires, Argentina

## Abstract

Current knowledge on the genetic basis of nonalcoholic fatty liver disease (NAFLD) suggests that variants contributing not only to the disease predisposition but histological severity as well are located in genes that regulate lipid metabolism. We explored the role of rs641738 C/T located in *TMC4* (transmembrane channel-like 4) exon 1 (p.Gly17Glu) and 500 bases- downstream of *MBOAT7* gene (*TMC4*/*MBOAT7)*, in the genetic risk for developing NAFLD in a case-control study. Our sample included 634 individuals (372 patients with NAFLD diagnosed by liver biopsy and 262 control subjects); genotyping was performed by a Taqman assay. Genotype frequencies in controls (CC: 84, CT: 137, TT: 41) and patients (CC: 134, CT: 178, TT: 60) were in Hardy-Weinberg equilibrium; minor allele frequency 40.8%. Our sample had 84–99% power if an additive genetic model is assumed for estimated odds ratios of 1.3–1.5, respectively. We found no evidence of association between rs641738 and either NAFLD (Cochran-Armitage test for trend, *p* = 0.529) or the disease severity (*p* = 0.61). Low levels of MBOAT7 protein expression were found in the liver of patients with NAFLD, which were unrelated to the rs641738 genotypes. In conclusion, the role of rs641738 in the pathogenesis of NAFLD is inconclusive.

## Introduction

Current understanding of the genetic basis of nonalcoholic fatty liver disease (NAFLD) suggests that variants contributing not only to the disease susceptibility but also histological severity are located in genes that regulate lipid metabolism. Specifically, the missense p.Ile148Met (rs738409) variant located in *PNPLA3* (patatin-like phospholipase domain-containing 3) has been consistently associated with increased liver fat content and NAFLD severity, including fibrosis, across different populations around the world^1–3^. The risk effect of rs738409 on developing NAFLD is the strongest ever reported for a common variant modifying the genetic susceptibility of the disease, representing ~5.3% of the total variance^2,3^ and a moderate odds ratio (OR) of NAFLD and NASH of ~ 3.3^3^. Likewise, the missense p.Glu167Lys (rs58542926) variant located in *TM*6*SF2* (transmembrane 6 superfamily member 2) gene, while protecting against cardiovascular disease (CVD)^4,5^, has been associated with a modest risk of liver fat accumulation (OR ~2.13)^5^, NAFLD, and the NAFLD severity^6–11^.

Interestingly, a missense (p.Gly17Glu, rs641738 C/T) variant located in exon 1 of *TMC4* (transmembrane channel-like 4) gene and intergenic downstream of *MBOAT7* gene has been associated with a modest risk of developing NAFLD (OR ~1.37), NASH, and fibrosis^12^. However, these findings were based on a large report involving patients of European descent^12^. Nonetheless, the authors observed that the effect of rs641738 was restricted to European-Caucasian individuals, while not being significant in African American and Hispanic population^12^. Unfortunately, the association of rs641738 and NAFLD could not be replicated in other populations around the world, including Europeans from different cohorts, except for a small study that included cases-only (*n* = 125)^13^. For instance, a recent study including a large sample (*n* = 515) of patients with NAFLD recruited from several centers across Germany showed that rs641738 was associated with a marginal effect on liver fibrosis (*p* = 0.046) without any effect on NAFLD or liver function test^14^. Similarly, results yielded by analyzing the data pertaining to a small cohort of patients that underwent bariatric surgery in two European centers failed to confirm any association of rs641738 and NAFLD^15^. Likewise, studies from Asia failed to find an association of the variant with NAFLD or NASH^16–18^.

**Figure 1:**
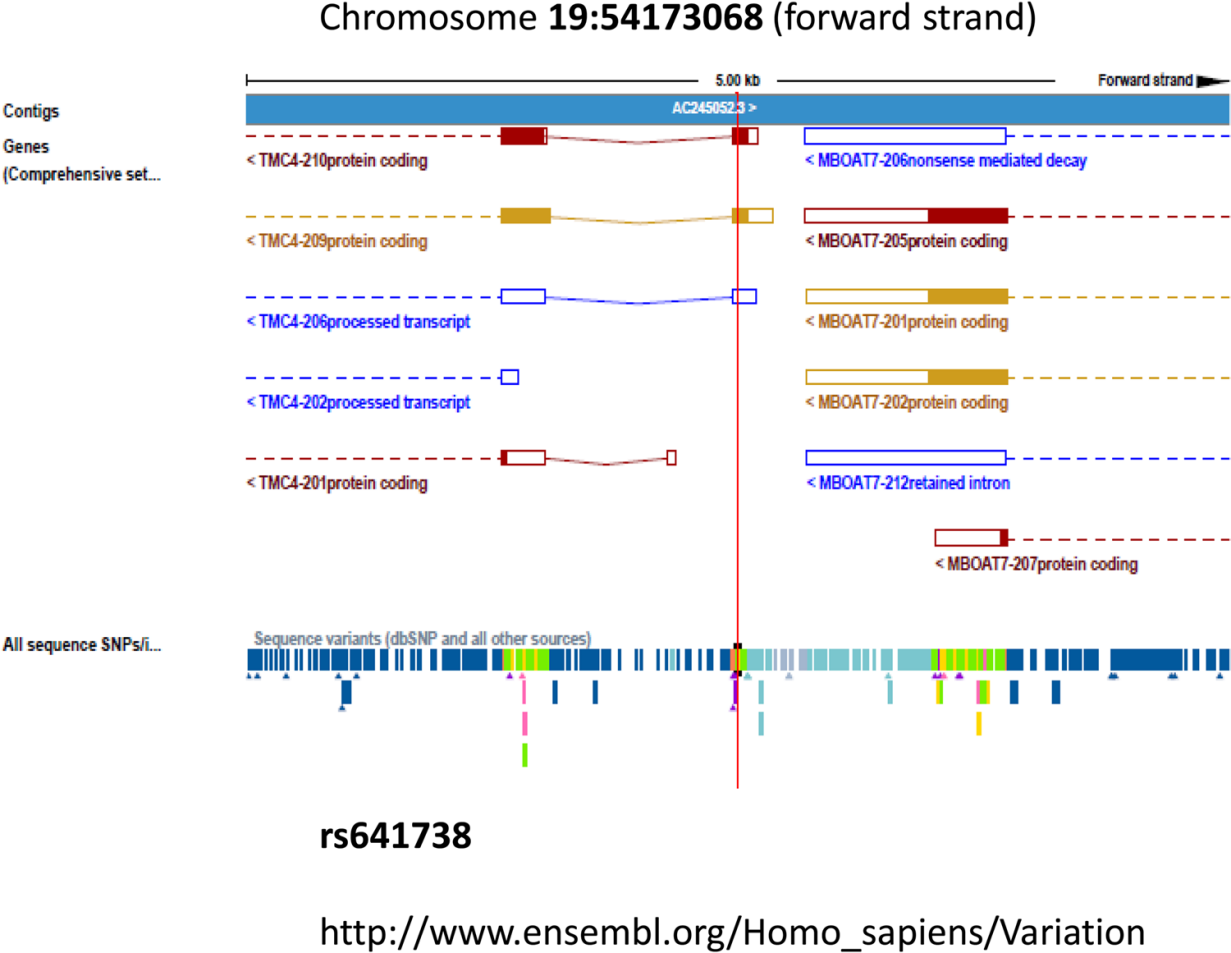
Genomic location of rs641738 in the forward strand of chromosome 19: 54,173,068. Figure shows the genomic assembly as a blue bar composed of individual contigs; rs641738 (outlined by a vertical red line) is shown in a 5 kb region along with surrounding variations. *TMC4* locus is located in chromosome 19: 54,160,168–54,173,171; *MBOAT7* locus is located in chromosome 19: 54,173,412–54,189,443.

The rs641738 variant is mapped 500 bases downstream of *MBOAT7* (membrane-bound O-acyltransferase domain-containing 7) locus (https://www.ncbi.nlm.nih.gov/snp/rs641738). Likewise, data from the genome assembly shows this variant located in exon 1 of the *TMC4* genomic region (19:54173068, GRCh38.p7 assembly) (Fig. 1). Annotation details provided by The Exome Aggregation Consortium (ExAC) (http://exac.broadinstitute.org/), shows that rs641738 as a transcript variant of *TMC4* locus. For that reason, previous reports refer to rs641738 as *MBOAT7* variant or *TMC4*/*MBOAT7* (https://www.ncbi.nlm.nih.gov/snp/rs641738).

In addition, speculations on the putative biological role of MBOAT7 in the pathogenesis of NAFLD still persist because the protein encoded by this gene is a lysophosphatidylinositol acyltransferase, which has specificity for arachidonoyl-CoA as an acyl donor.

Combined, available evidence suggests that associations of rs641738 with NAFLD and NASH remain to be either confirmed or refuted. Hence, we performed a hospital-based case-control study to explore the association between rs641738 and NAFLD, including adult patients in whom the histological disease severity was confirmed by liver biopsy.

In addition, we explored the protein expression pattern of MBOAT7 in the liver of patients with NAFLD to provide evidence of whether the protein encoded by this locus might be involved in the biology of the disease.

## Results

### The rs641738 is not associated with NAFLD or the histological disease severity

Clinical and biochemical features of patients and controls are disclosed in Tables 1 and 2.

**Table 1:** Clinical and biochemical features of patients and controls in the cross-sectional study of patients with NAFLD and Metabolic Syndrome (MetS).

Variables	Control subjects	NAFL	NASH
Number of subjects	241	113	153
Female, %	46	56	67
Age, years	47 ± 14	54 ± 0.8	50 ± 0.9
BMI, kg/m^2^	25 ± 4.2	31.5 ± 5.4^#^	34 ± 6.0^+,*^
Fasting plasma glucose, mg/dL	84 ± 15	97 ± 22^#^	129 ± 119^+,*^
Fasting plasma insulin, μU/ml	6.8 ± 4.7	13 ± 9^#^	16 ± 11^+,*^
HOMA-IR index	1.4 ± 1.0	3 ± 2.1^#^	5.1 ± 6.6^+,*^
Total cholesterol, mg/dL	205 ± 42	207 ± 53	211 ± 43
HDL-cholesterol, mg/dL	57 ± 16	53 ± 25	50 ± 14
LDL-cholesterol, mg/dL	123 ± 38	126 ± 48	125 ± 42
Triglycerides, mg/dL	120 ± 77	154 ± 76^#^	192 ± 119^+^
ALT, U/L	20 ± 6.0	56 ± 60^#^	73 ± 54^+*^
AST, U/L	17.5 ± 6.6	35 ± 17^#^	51 ± 33^+*^
**Histological Features**			
Degree of steatosis, %	—	47 ± 25	61 ± 21*
Lobular inflammation (0–3)	—	0.6 ± 0.6	1.2 ± 0.6*
Hepatocellular ballooning (0–2)	—	0.03 ± 0.19	0.9 ± 0.6*
Fibrosis Stage	—	0.03 ± 0.3	1.4 ± 1.24*
NAFLD activity score (NAS)	—	2.6 ± 1.1	4.5 ± 1.4*

NAFL: nonalcoholic fatty liver, NASH: nonalcoholic steatohepatitis BMI: body mass index; HOMA: homeostatic model assessment; ALT and AST: Serum alanine and aspartate aminotransferase. Results are expressed as mean ± SD.

^#^p < 0.001 Indicates NAFL vs. controls, ^*^p < 0.001 indicates comparisons between NAFL and NASH, and ^+^p < 0.001 denotes comparisons between NASH and control subjects.

P value stands for statistical significance using Mann-Whitney *U* test, except for female/male proportion that p value stands for statistical significance using Chi*-*square test.

**Table 2:** Clinical and biochemical features of morbid obese patients recruited from the bariatric surgery cohort.

Variables	Control patients (morbid obese-no NAFLD)	NAFL	NASH
Number of subjects	21	54	52
Female, %	75	67	59
Age, years	38.2 ± 10	39 ± 9	46 ± 12
BMI, kg/m^2^	53 ± 11	53 ± 13	48 ± 12
Fasting plasma glucose, mg/dL	95.5 ± 13.4	105 ± 30^#^	146 ± 76^+,*^
Fasting plasma insulin, μU/ml	12 ± 5.5	13 ± 6.5^#^	33 ± 56^+,*^
HOMA-IR index	2.64 ± 1.4	3.2 ± 1.6^#^	19.6 ± 52^+,*^
Total cholesterol, mg/dL	187 ± 37	185 ± 35	197 ± 54
HDL-cholesterol, mg/dL	40 ± 9	45 ± 10	41 ± 6.7
LDL-cholesterol, mg/dL	122 ± 32	124 ± 27	135 ± 47
Triglycerides, mg/dL	130 ± 57	149 ± 54	200 ± 119^+^
ALT, U/L	21 ± 6	34 ± 25^#^	40 ± 18^+^
AST, U/L	15.7 ± 5	26 ± 18^#^	30 ± 13^+^
**Histological Features**			
Degree of steatosis, %	—	36.5 ± 26	51 ± 27^*^
Lobular inflammation (0–3)	—	0.3 ± 0.54	1.5 ± 0.96^*^
Hepatocellular ballooning (0–2)	—	0.20 ± 0.4	1.1 ± 0.6^*^
Fibrosis Stage	—	0.3 ± 1.67	1.5 ± 1.4^*^
NAFLD activity score (NAS)	—	2.2 ± 1.37	4.8 ± 1^*^

NAFL: nonalcoholic fatty liver, NASH: nonalcoholic steatohepatitis BMI: body mass index; HOMA: homeostatic model assessment; ALT and AST: Serum alanine and aspartate aminotransferase. Results are expressed as mean ± SD.

^#^p < 0.001 Indicates NAFL vs. controls, ^*^p < 0.001 indicates comparisons between NAFL and NASH, and ^+^p < 0.001 denotes comparisons between NASH and control subjects.

P value stands for statistical significance using Mann-Whitney *U* test, except for female/male proportion that p value stands for statistical significance using Chi*-*square test.

Genotype frequencies in controls (*n* = CC: 84, CT: 137, TT: 41, *p* = 0.22) and patients (CC: 134, CT: 178, TT: 60, *p* = 0.94) were in Hardy-Weinberg equilibrium (HWE). The minor allele frequency (MAF) in our sample was 40.8%, in line with that reported in the 1000 Genomes Project for the T allele in all populations (37%) and Europeans (44%) (1000 Genomes Project, Phase 3, http://www.ensembl.org).

The association analysis of rs641738 and NAFLD showed no effect of the variant on the susceptibility of NAFLD (Cochran-Armitage test for trend χ^2^ = 0.397, *p* = 0.529). The variant was associated with neither NASH nor the disease severity (*p* = 0.61). No association was found with fibrosis status (fibrosis yes/no) (*p* = 0.95), lobular inflammation (*p* = 0.46), or NAFLD- NAS score (*p* = 0.25). However, in univariate analysis we observed a significant association with circulating triglycerides (TG) (*p* = 0.004). The rs641738 was not associated with glucose metabolism, HOMA-index, total, HDL, LDL-cholesterol or other MetS components.

**Figure 2:**
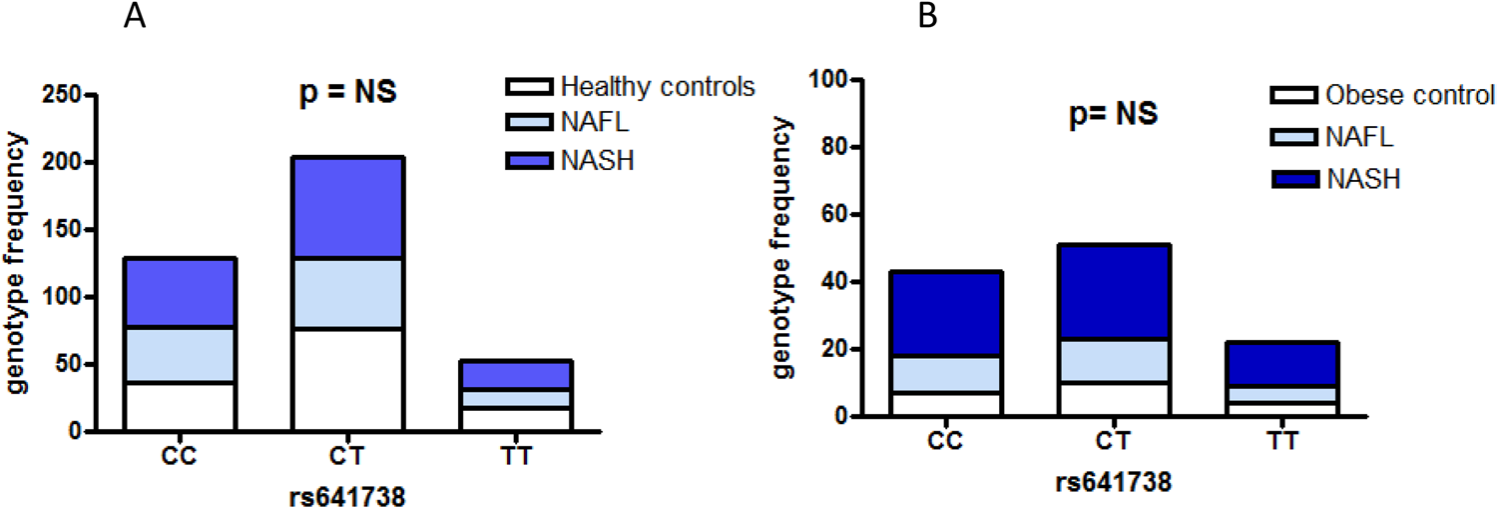
Genotype frequencies of rs641738 according the disease status (control subjects, patients with simple steatosis-NAFL and NASH). (**A**) Results from the cross-sectional study of patients with NAFLD and Metabolic Syndrome. (**B**) Results from a cohort-study of morbid-obese patients that underwent bariatric surgery.

Genotype frequencies of rs641738 according to the disease status (control subjects, patients with simple steatosis-NAFL and NASH) in the two studied groups are shown in Fig. 2A,B.

### MBOAT7 is expressed in the liver of patients with NAFLD at low levels

In order to provide evidence supporting a putative role of *MBOAT7* in the biology of NAFLD, we further explored whether the protein encoded by this gene is expressed in the liver.

**Figure 3:**
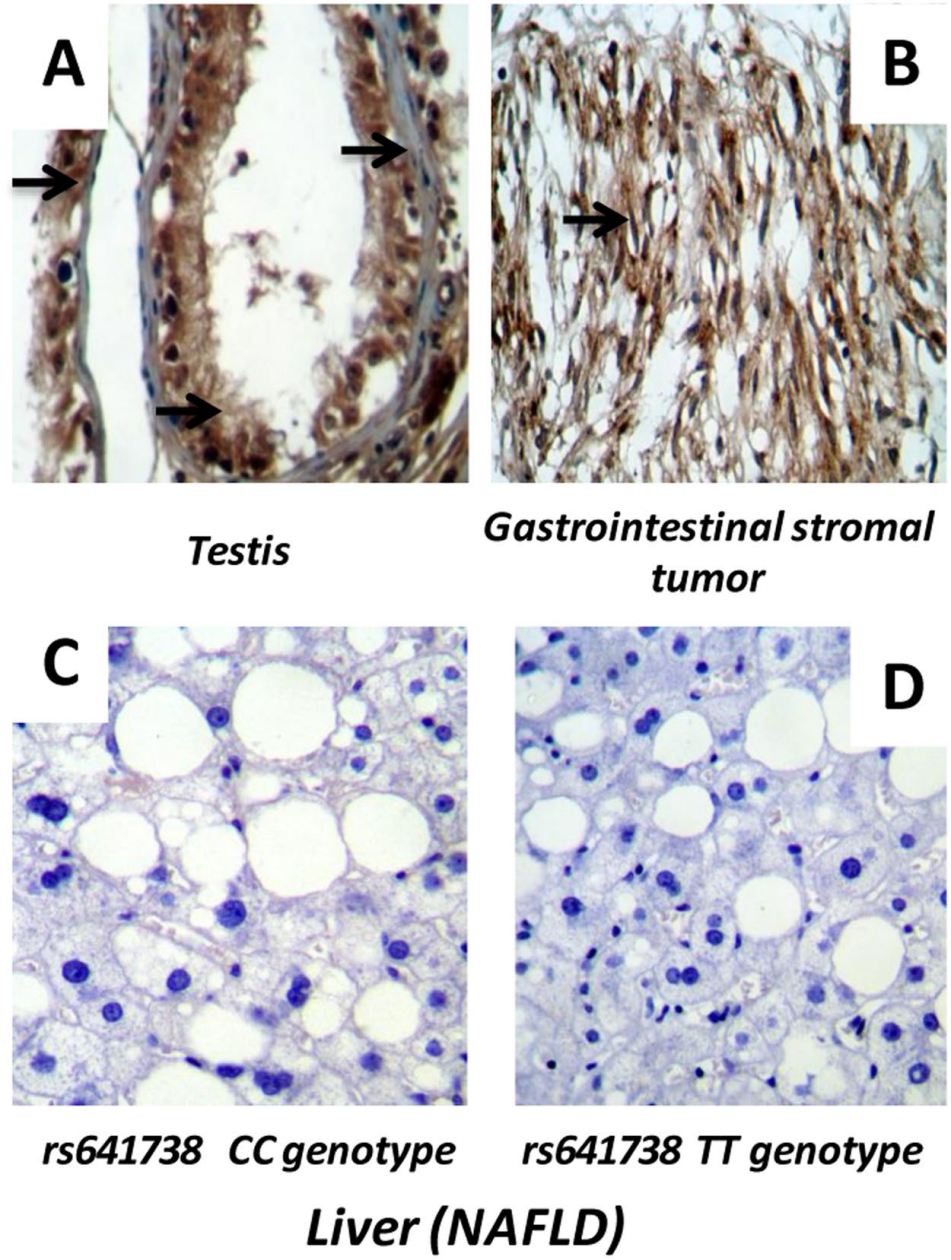
Expression profile of MBOAT7 in the liver tissue of patients with NAFLD. (**A**) A representative sample of testis and (**B**) A representative sample of gastrointestinal stromal tumor, which were used as positive control tissues; arrows denote strong immunoreactivity. (**C**) and (**D**) A representative sample of a patient with NAFLD carrying the rs641738 CC and TT genotype, respectively. Protein expression was assessed by imunohistochemistry in ten patients with NAFLD (CC *n* = 5 vs. TT *n* = 5) by two independent Pathologists and a semiquantitative score (0–4). As the samples presented very low levels of staining no sample was classified as having an score higher than 1. Mann-Whitney U test was used to analyse statistical significance.

As positive control tissues, we included a sample of testis and a sample of gastrointestinal stromal tumor retrieved from the collection of our Pathology Department in which we observed a strong immunoreactivity of MBOAT7 (Fig. 3A,B). In contrast, in the liver of patients with NAFLD, we found very low expression levels of the protein assessed by immunohistochemistry (Fig. 3C,D). Thus, our results are comparable to the information displayed in the Human Protein Atlas (http://www.proteinatlas.org/ENSG00000125505-MBOAT7/tissue). Furthermore, we found no differences in the liver MBOAT7 expression pattern between rs641738 genotypes (CC 0.8 ± 0.27 vs. TT 0.9 ± 0.22, *p* = 0.69) (Fig. 3C,D).

## Discussion

In this study, we explored the role of the missense rs641738 variant in the susceptibility of NAFLD and the disease severity. We did not find statistically significant differences in genotypic or allelic frequencies for the variant in either the predisposition of NAFLD or NASH, or other related histological features. Genotype frequencies in controls and cases were in HWE, and sample size estimation showed at least 84% power for the additive genetic model even if a very modest effect (OR: 1.3) is considered. Power calculation based on a OR of 1.3 is justified by previous evidence of association of the variant and NAFLD (OR 1.37)^12^ or liver fibrosis (OR 1.41)^12^ in European American population; in fact, our entire sample is composed of individuals of self-reported European ancestry. In addition, for polymorphisms with minor allele frequencies >0.2 (like the one observed for rs641738 MAF 0.40), the ORs are expected be in the range of 1.1–1.5^19^. However, our study is underpowered for ORs 1.2–1.25 and for all histological features analyses.

In contrast to some reports in the literature indicating a significant association of the variant with NASH, liver damage and fibrosis in individuals of European descent but not other ethnicities^12^, our study suggests that it is highly unlikely that rs641738 plays a role in the genetic susceptibility of NAFLD, at least in our population. A note of caution regarding the lack of association of the variant and liver fibrosis in our sample should be added because it could be explained by insufficient power.

Likewise, Krawczyk and coworkers failed to detect an association of the rs641738 and NAFLD or liver function test^14^, while a marginal but positive effect of the variant on liver fibrosis (OR 1.41 95% CI 1.003–1.982, *p* = 0.048) was observed.

Meanwhile this manuscript was under the peer review process, several reports on the role of rs641738 were published^16–18,20^; specifically, there were large studies that included well characterized patients diagnosed by liver biopsy^16,17^. Interestingly, these studies showed a negative association of the variant with NAFLD^16–18,20^ and NASH or liver fibrosis^16,17^.

A detailed summary of the available evidence is shown in Table 3.

**Table 3:** A summary of studies that explored the association between the rs641738 and NAFLD, liver histology, and related traits.

First author, Year, Reference	Country, Ancestry	Study design	Sample size and features	Association with NAFLD as disease trait	Effect on liver histology and liver enzymes
**Studies that reported lack of association of rs641738 and NAFLD**					
Current study (Sookoian S, 2018	Argentina, Caucasian descent	Case-control hospital based	634 individuals (372 patients with NAFLD diagnosed by liver biopsy and 262 control subjects)	No evidence of association	No evidence of association with NASH, fibrosis, or liver enzymes
Lin YC, 2018^18^	Taiwan, Han Chinese population	Population based; 189 (22.7%) had hepatic steatosis	831 obese children aged 7–15 years. NAFLD diagnosed by liver ultrasonographic examination	No evidence of association	No evidence of association with liver enzymes
Koo BK, 2017^17^	Korea, Asian descent.	Case-control hospital based	525 individuals (416 patients with NAFLD diagnosed by liver biopsy and 109 healthy controls)	No evidence of association	No evidence of association with NASH, fibrosis, or liver enzymes
Dold L, 2017^20^	Germany, Caucasian descent	Case-control hospital based	291 individuals (142 HIV-infected patients and 149 healthy blood donors). Liver stiffness assessed by Fibroscan and NAFLD by liver ultrasound.	No evidence of association	No evidence of association with liver enzymes or liver stiffness
Krawczyk M, 2017^14^	Germany, Caucasian descent	Cases only	515 patients with NAFLD. Liver biopsy was performed in a sub-group of 320 patients.	No evidence of association	Association with liver fibrosis (*P* = 0.046)
Krawczyk M, 2016^15^	Germany, Caucasian descent	Cases only	84 obese individuals scheduled for bariatric surgery	No evidence of association	No evidence of association with NASH, fibrosis, or liver enzymes
Kawaguchi T, 2018^16^	Japan, Asian descent	Case-control hospital based	8608 individuals (936 histologically proven NAFLD patients and 7,672 general-population controls)	No evidence of association with hepatic steatosis	No evidence of association with NASH, fibrosis, or liver enzymes
**Studies that reported association of rs641738 and NAFLD and NASH**					
Mancina R, 2016^12^	Multi-ethnic	Two stages. First stage: population based. Second stage: cases-only, hospital based	3854 participants from the Dallas Heart Study (a multi-ethnic population-based sample of Dallas County residents; hepatic triglyceride content evaluated by liver spectroscopy n = 2736) and 1149 European individuals (NAFLD evaluated by liver biopsy).	First stage: association with NAFLD in the global analysis. Stratification by ethnic groups: association only significant in African Americans but not European)	Association with hepatic steatosis (p = 0.015), NASH (p = 0.05), and fibrosis stage F2-4 (p = 0.012).
Luukkonen P, 2016^13^	Finland	Cases only	125 patients with NAFLD assessed by liver biopsy	Not assessed	Association with hepatic steatosis degree (p = 0.03), inflammation (p = 0.04) and fibrosis stage F2-4 (p = 0.01)
Viitasalo A, 2016^31^	Finland	Population based	467 Caucasian children aged 6–9 years, *no assessment of NAFLD by any imaging method.	Not assessed	Association with plasma ALT levels (additional adjustment for body fat percentage show not association with liver enzymes (*P* = 0.063)

While the reasons behind these discrepancies are unclear, several potential explanations should be considered.

The first explanation relates to putative discrepancies at the population level and the design of the extant studies on the effect of rs641738 on either hepatic steatosis or hepatic triglyceride content (HTGC)—as measured by liver spectroscopy—both of which contribute to inconsistencies among different datasets. For example, in their analyses, Mancina *et al*. stratified the data by ethnic groups of the population-based Dallas Heart Study (DHS), and observed a positive significant effect (*p* = 0.019) of rs641738 on HTGC content (continuous variable) that was restricted to African Americans. In contrast, association with hepatic steatosis (NAFLD as a disease trait) remained significant in European Americans (OR: 1.37; 95% CI: 1.09–1.72; *p* = 0.007) but not in African Americans^12^. The biological reasons behind such discrepancies, while interesting, are certainly hard to explain.

A second explanation could be a false positive association between the variant and NAFLD ascribed to deviations from HWE or insufficient genotyping accuracy. Mancina *et al*. showed that rs641738 was associated with NASH and the disease severity in European population; however, genotype frequencies deviated from HWE (*p* = 0.017)^12^. Nevertheless, HWE is statistically a null hypothesis as it assumes there is no evolution in the population. In fact, disease-associated allele can be deviated from HWE in a disease population (cases) but not in controls.

A third but yet unexplored explanation relates to a putative gene × environment interaction, the occurrence of which seems to be limited to the European cohort of Mancina *et al*.’s study^12^. Nevertheless, this possibility is hard to conceive, as the variant was not associated with NAFLD in patients from other European countries, including Germany^14,15^; although the results of the German study could be a remote example of this situation.

In our sample we observed that rs641738 was associated with triglyceride levels and so, it might indirectly regulate intermediate steps of fatty acid (polyunsaturated fatty acids-PUFA and PUFA-containing TG) biosynthesis. Still, the association of the variant with TG was not observed among individuals included in previous reports^12^, except for one study from Germany^15^; hence, the biological meaning of this observation remains unknown.

Moreover, a distant but noteworthy explanation could pertain to disparities in the minor allele frequency (MAF) among different populations around the world, including our sample. However, the frequency of the risk allele in our population is comparable to that reported in the 1000 Genomes Project for Caucasians (44%); hence, it seems unlikely that discrepancies among studies arise from racial differences. It is still possible, however, that this variant may bare a population-specific association with NAFLD. It is also likely that rs641738 may work via an interaction with other injuries or unknown environmental factors, especially when these factors may be distributed differently among different populations.

**Figure 4:**
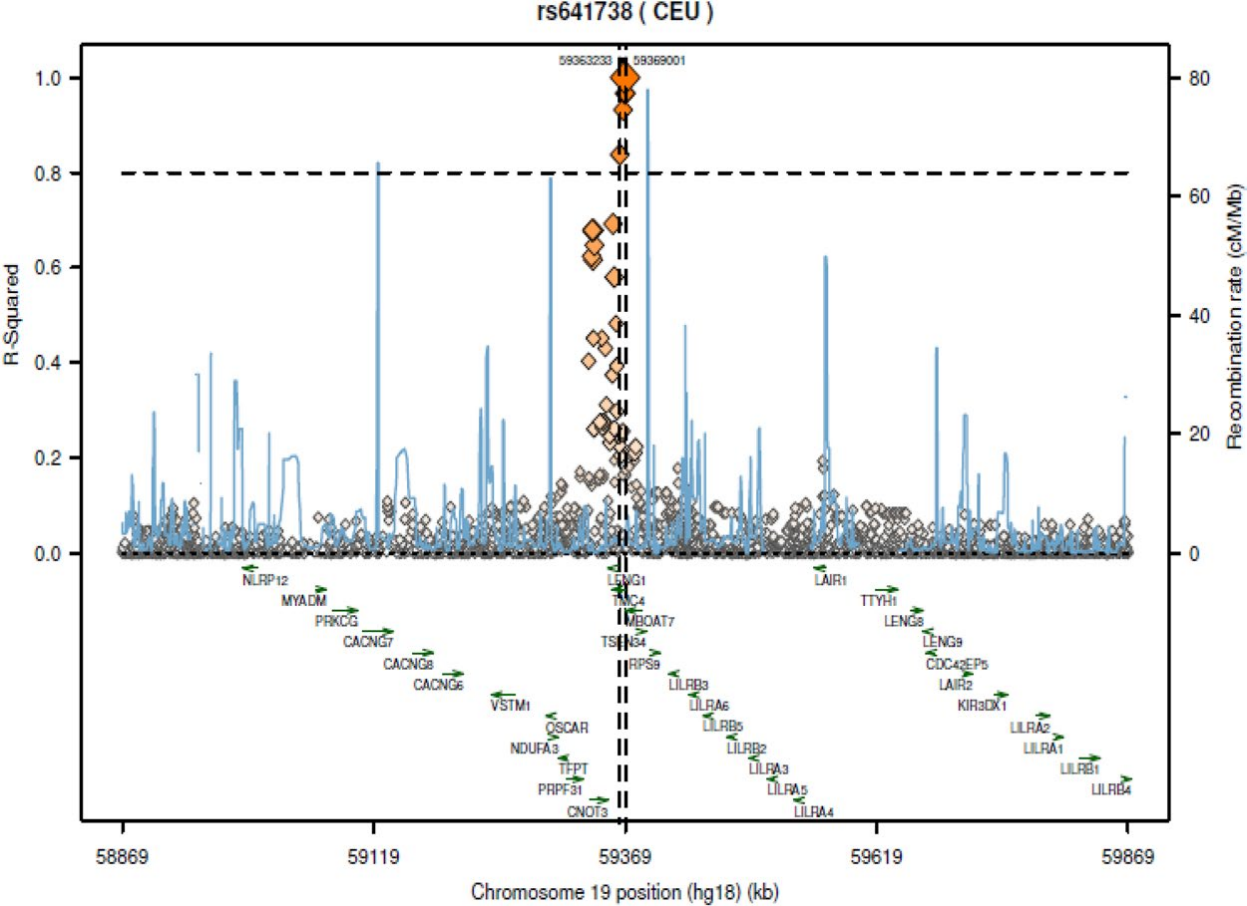
Exploration of potential regulatory variants in linkage disequilibrium with rs641738. Plot was retrieved from *SNAP*, a web-based tool for identification and annotation of proxy SNPs (https://personal.broadinstitute.org/plin/snap/index.php). SNAP finds proxy SNPs based on linkage disequilibrium and physical distance. Pair-wise linkage disequilibrium is pre-calculated based on phased genotype data from the International HapMap Project. The plot shows the associated region (chromosome 19, rs641738), defined by the contiguous region that contains all proxy SNPs with *r*^2^ > 0.8.

A final plausible explanation is that rs641738 is not necessarily the causal variant; thus, other SNPs in strong linkage disequilibrium (LD) could explain an impact on the phenotype. Exploration of variants in high LD with rs641738 shows at least five SNPs, (Fig. 4 and Table 4), including rs8736 a 3′ UTR variant of *MBOAT7/*2 kb upstream variant of *TMC4*.

**Table 4:** A description of variants in strong linkage disequilibrium (LD) with rs641738, including potential functionality and location in locus (variant feature).

**Proxy**	**Distance**	**R** ^**2**^	**D Prime**	**Chr**	Locus*	Variant feature*
**rs641738**	—	—	—	chr19	***MBOAT7/TMC4***	TMC4: Missense Variant/MBOAT7: 500B Downstream Variant
rs626283	238	0.967	1	chr19	***MBOAT7/TMC4***	TMC4: 2KB Upstream Variant/MBOAT7: 500B Downstream Variant
rs2576452	330	0.967	1	chr19	***TMC4***	Intron Variant
rs8736	426	0.967	1	chr19	***MBOAT7/TMC4***	TMC4: Upstream Variant/MBOAT7: 3 Prime UTR Variant2KB
rs4806498	2021	0.932	0.966	chr19	***TMC4***	Intron variant
rs60204587	5342	0.838	0.963	chr19	***TMC4***	Intron variant

Chr: chromosome, UTR: untranslated region.

^*^https://www.ncbi.nlm.nih.gov/snp (GRCh38.p7).

As a final point, we would like to comment on the significant dissimilarities that indeed exist between the biological function of *MBOAT7* and *TMC4*. In fact, while MBOAT7 is a protein involved in the pathway of phospholipid metabolism, TMC4 is involved in the transport of ions. More specifically, *MBOAT7* encodes a member of the membrane-bound O-acyltransferases family of integral membrane proteins that exhibit acyltransferase activity. The encoded protein is a lysophosphatidylinositol acyltransferase that has specificity for arachidonoyl-CoA as an acyl donor; this protein is involved in the reacylation of phospholipids as a part of the phospholipid remodeling pathway known as the Land cycle.

Analysis of eQTLs (expression quantitative trait loci), which denote correlations between genotype and tissue-specific gene expression levels, shows that rs641738 is associated with eQTLs in the liver (Tables 5 and 6) and other tissues and cell types as well (Table 6). Likewise, other *MBOAT7-*variants, including some that are in strong LD with rs641738, are associated with many eQTLs in the liver tissue (Table 5). Complete details of significant Single-Tissue eQTLs for *MBOAT7* (ENSG00000125505.12) and *TMC4 (ENSG00000*16*7608.7)* in the liver tissue are shown in Table 5.

**Table 5:** Significant Single-Tissue eQTLs for *MBOAT7* (ENSG00000125505.12) and *TMC4 (ENSG00000167608.7)* in the liver tissue.

**Query Gencode Id**	**SNP Id**	**P-Value**	**Effect Size**
***MBOAT7*** **(ENSG00000125505.12)**			
ENSG00000125505.12	rs11668882	8.80E-10	−0.43
ENSG00000125505.12	rs8736	1.00E-09	−0.43
ENSG00000125505.12	rs2576452	1.10E-09	−0.43
ENSG00000125505.12	rs372932354	1.40E-09	−0.42
ENSG00000125505.12	**rs641738**	**2.80E-09**	**−0.42**
ENSG00000125505.12	rs626283	2.80E-09	−0.42
ENSG00000125505.12	rs4806498	3.60E-09	−0.42
ENSG00000125505.12	rs60204587	4.10E-09	−0.43
ENSG00000125505.12	rs10416555	5.90E-08	−0.61
ENSG00000125505.12	rs36656	0.0000011	−0.34
ENSG00000125505.12	rs77215230	0.0000012	−0.6
ENSG00000125505.12	rs1050527	0.0000012	−0.6
ENSG00000125505.12	rs11084313	0.0000018	−0.58
ENSG00000125505.12	rs8100978	0.0000043	−0.36
***TMC4 (ENSG00000167608.7)***			
ENSG00000167608.7	rs776138589	6.80E-20	1.6
ENSG00000167608.7	rs43211	2.80E-08	−0.67
ENSG00000167608.7	rs117643023	3.60E-08	1.4
ENSG00000167608.7	rs4806716	6.20E-08	−0.67
ENSG00000167608.7	rs8100978	1.30E-07	−0.63
ENSG00000167608.7	rs36663	1.40E-07	−0.65
ENSG00000167608.7	rs36659	1.40E-07	−0.65
ENSG00000167608.7	rs42319	2.10E-07	−0.64
ENSG00000167608.7	rs36642	2.20E-07	−0.65
ENSG00000167608.7	rs36641	2.30E-07	−0.64
ENSG00000167608.7	rs593073	3.80E-07	−0.64
ENSG00000167608.7	rs3816129	3.90E-07	−0.63
ENSG00000167608.7	rs36658	4.10E-07	−0.64
ENSG00000167608.7	rs8101186	4.40E-07	−0.6
ENSG00000167608.7	rs653560	4.60E-07	−0.64
ENSG00000167608.7	rs40168	6.90E-07	−0.61
ENSG00000167608.7	rs36624	8.10E-07	−0.59
ENSG00000167608.7	rs40167	0.000001	−0.6
ENSG00000167608.7	rs635608	0.0000012	−0.6
ENSG00000167608.7	rs7595	0.0000012	−0.6
ENSG00000167608.7	rs36621	0.0000012	−0.6
ENSG00000167608.7	rs12975696	0.0000013	−0.59
ENSG00000167608.7	rs40357	0.000002	−0.59
ENSG00000167608.7	rs183716	0.000002	−0.59
ENSG00000167608.7	rs39714	0.000002	−0.59
ENSG00000167608.7	rs36623	0.000002	−0.59
ENSG00000167608.7	rs36622	0.0000027	−0.6

eQTL: expression quantitative trait loci. Data Source: The Genotype-Tissue Expression (GTEx) project (Data Source: GTEx Analysis Release V7 (dbGaP Accession phs000424.v7.p2). The eQTL effect allele is the alternative allele relative to the reference allele in the human genome reference, not the minor allele. Query was specifically done on MBOAT7 locus (ENSG00000125505.12).

**Table 6:** Analysis of eQTLs (expression quantitative trait loci) denoting correlations between rs641738 and cell tissue-specific gene expression levels.

**Query SNP**	**LD-eQTL**	**exGene**	**Association**	**LD**	**Study**
rs641738	rs641738	TMC4	6.00E-11	1	LV: Caucasian liver donors
rs641738	rs641738	MBOAT7	3.65E-12	1	LV: Caucasian liver donors
rs641738	rs641738	TFPT	3.88E-03	1	LV: Caucasian liver donors
rs641738	rs641738	MBOAT7	9.364	1	LV2: Liver donors
rs641738	rs641738	TMC4	2.001e-08	1	EGEUV_EUR: 1000 Genome-EUR
rs641738	rs2576452	TMC4	7.283e-09	0.923	MuTHER_Fat
rs641738	rs626283	TMC4	6.631e-09	1	MuTHER_Fat
rs641738	rs641738	TMC4	7.858e-09	1	MuTHER_Fat
rs641738	rs8736	TMC4	5.979e-09	0.92	MuTHER_Fat
rs641738	rs2576452	TMC4	1.234e-19	0.923	MuTHER_Skin
rs641738	rs626283	TMC4	3.031e-20	1	MuTHER_Skin
rs641738	rs641738	TMC4	5.080e-20	1	MuTHER_Skin
rs641738	rs8736	TMC4	1.052e-19	0.92	MuTHER_Skin

Table shows tissue-specific eQTL associations were identified by comparing eQTL data from six cell types: LCLs, B cells, Monocytes, Brain, Liver, and Skin.

Data was extracted from the integrated eQTL database, which is available at: http://www.exsnp.org/LDeQTL. Query was specifically done on rs641638.

All eQTL association data in this database were collected from 16 publicly available studies that had been performed on various human tissues and populations.

MuTHER: Multiple Tissue Human Expression Resource.

The observation that rs641738 is associated with eQTLs in non-liver tissues, including fat, might reinforce the possibility of unexplored associations between the variant and the disease. For example, a recent study showed that associations between common gene variants and NAFLD are uncovered by adiposity degree^21^; this point could explain some of the above mentioned observations.

Information regarding protein expression is much limited. We have specifically assessed the protein expression pattern of MBOAT7 in the liver of patients with NAFLD and we observed no evidence of robust immunoreactivity. Similarly, we failed to observe any association between liver-MBOAT7 expression and rs641738 genotypes (CC, TC and TT) (Fig. 3). Contrasting evidence was published elsewhere suggesting that rs641738 T allele was associated with reduced mRNA and hepatic protein MBOAT7 expression in patients with advanced fibrosis^12,22^. Then, a putative yet uncovered *in cis* effect of rs641738 (i.e., pertaining to mRNA stability or translation) might explain participation of the variant in lipid metabolism by regulating MBOAT7 expression.

On the other hand, *TMC4* encodes for a membrane protein involved in the transport channel expressed in the peripheral nervous system; TMC4 belongs to the calcium-dependent chloride channel (ca-clc) family highly expressed among epithelia (kidney, small intestine, colon)^23^. Interestingly, there is evidence supporting the presence of chloride channels not only in the plasma membrane of hepatocytes but in multiple intracellular compartments as well^24^. The involvement of ion channels in the pathogenesis of NAFLD and/or in pathways associated with hepatic fibrogenesis remains elusive; nevertheless, it certainly represents an interesting path for future research. In fact, this observation could explain previously reported associations of rs641738 and fibrosis in patients with chronic hepatitis C and B^25,26^, and NAFLD^12–14^. Still, the exact effect/mechanisms by which the variant could regulate liver fibrogenesis remains uncertain. We reinforce the importance of precision in identifying the genomic location and the biological function of a given variant, as this would increase not only the understanding of the genetic component of NAFLD but also its relationship with the disease pathogenesis.

In conclusion, the rs641738 is not associated with NAFLD in our population. The association of the variant and NAFLD as disease trait could not be replicated in population-based or hospital based studies from Asia^16–18,20^ or Germany^14^. Nevertheless, the association with liver histology, including fibrosis was only observed in patients with NAFLD of European ancestry^12–14^; this finding could not be replicated in studies that included Asian population^16,17^. Hence, larger studies are required before any definitive conclusion can be reached.

## Patients and Methods

### Patients and control subjects: selection criteria

The study included a sample of 634 unrelated individuals, of which 262 were controls subjects and 372 were patients who have histopathologic-proven features of NAFLD. Patients and controls were selected from two different hospital-based settings, including a cross-sectional study of patients diagnosed with NAFLD and Metabolic Syndrome (MetS) in the Liver Unit, Hospital Abel Zubizarreta, Buenos Aires, Argentina, and an independent cohort of morbid-obese patients that underwent bariatric surgery in the Surgery Department, Hospital de Alta Complejidad en Red El Cruce, Buenos Aires, Argentina.

All investigations performed were conducted in accordance with the guidelines of the 1975 Declaration of Helsinki. Informed and written consent for study participation from all individuals was obtained in accordance with the procedures approved by the ethical committee of our institution (protocol number: 104/HGAZ/09, 89/100 and 1204/2012).

Exclusion criteria: Secondary causes of steatosis, including alcohol abuse (≥30 g alcohol daily for men and ≥20 g for women), total parenteral nutrition, hepatitis B and hepatitis C virus infection, and the use of drugs known to precipitate steatosis were excluded. In addition, patients with any of the following diseases were excluded from participation: autoimmune liver disease, metabolic liver disease, Wilson’s disease, and α-1-antitrypsin deficiency.

Control subjects that matched patients with NAFLD-MetS were selected from subjects attending our hospital for check-up purposes whose age and sex matched the NAFLD patients. In addition to the standard heath examination, all non-obese control individuals were subjected to a liver ultrasonographic (US) examination. They were included in the study if they did not have evidence of fatty change or biochemical abnormalities. Furthermore, control subjects were confirmed not to have any of the features of the metabolic syndrome as defined by the National Cholesterol Education Program Adult Treatment Panel III and did not abuse alcohol.

In the population of morbid obese patients, control subjects were obese patients who also underwent bariatric surgery and had not features of NAFLD demonstrated in the liver biopsy.

The case participants and the controls were selected during the same study period from the same population of patients attending the above mentioned institution, and all of them share the same demographic characteristics (occupation, educational level, place of residence, and ethnicity).

### Physical, anthropometric, biochemical evaluation and histological

Health examinations included anthropometric measurements, a questionnaire on health-related behaviours and biochemical determinations.

The disease severity was assessed by liver biopsy that was performed before any intervention with ultrasound guidance and a modified 1.4-mm-diameter Menghini needle (Hepafix, Braun, Germany) under local anesthesia on an outpatient basis or during bariatric surgery. All liver biopsies were evaluated by the same pathologist.

A portion of each liver biopsy specimen was routinely fixed in 40 g/l formaldehyde (pH 7.4), embedded in paraffin, and stained with hematoxylin and eosin, Masson trichrome, and silver impregnation for reticular fibers. All the biopsies were at least 3 cm in length and contained a minimum of 8 portal tracts. The degree of steatosis was assessed according to the system developed by Kleiner *et al*., based on the percentage of hepatocytes containing macrovesicular fat droplets^27^. NASH and NAFLD Activity Score (NAS)^27,28^ were defined as reported previously; a NAS threshold of 5 was used for further comparisons with variables of interest, NASH was defined as steatosis plus mixed inflammatory-cell infiltration, hepatocyte ballooning and necrosis, Mallory’s hyaline, and any stage of fibrosis, including absent fibrosis^27,28^.

### Genotype and association analysis, and power and sample size calculation

The genetic analyses were done on genomic DNA extracted from white blood cells. Genotyping of rs641738 was performed using a TaqMan genotyping assay (dbSNP rs641738 assay C___8716820_10, # 4351379; Applied Biosystems, California 92008, USA) according to manufacturer’s instructions. To ensure genotyping quality, we included DNA samples as internal controls, hidden samples of known genotype, and negative controls (water). The overall genotype completion rate was 100%.

To account for possible population stratification, we used a collection of 13 SNPs at different loci (located in chromosomes 4, 15, 17, 13, 1, and 3) and then analyzed the data with the Structure program Version 2^29^ as we explained elsewhere^2^. We found no evidence of stratification in our sample because the cases and the controls showed similar Q values and the Structure program assigned a similar distance to clusters with no further improvement in the fitting model by adding up to four clusters (the ln of likelihood was maximum for K = 1). Moreover, all the participants in this study self-reported a Caucasian ethnicity as a surrogate of ancestry, which is consistent with the observed MAF.

Using the CaTS power calculator for genetic association studies^30^ and assuming a prevalence of NAFLD of 0.30, minor allele frequency (MAF) T = 0.40 and an odds ratio (OR) of 1.3–1.5, our sample had 84–99% power, respectively, for the additive genetic model.

### Liver Immunohistochemistry

Four-micrometer sections were mounted onto silane coated glass slides to ensure section adhesion through subsequent staining procedures. Briefly, sections were deparaffinized, rehydrated, washed in phosphate buffer solution (PBS), and treated with 3% H_2_O_2_ in PBS for 20 min at room temperature to block endogenous peroxidase. Following microwave heat-induced epitope retrieval in 0.1 M citrate buffer at pH 6.0 for 20 min, the slides were incubated with a dilution of 1:100 of rabbit polyclonal antibody for Human Anti-MBOAT7 (ARP49811_T100, Aviva Systems Biology, San Diego, CA 92121 USA). Immunostaining was performed using the VECTASTAIN Elite ABC Kit (Vector Lab. CA, USA) detection system. Subsequently, slides were immersed in a 0.05% 3,3′-diaminobenzidine solution in 0.1 M Tris buffer, pH 7.2, containing 0.01% H_2_O_2_. After a brown color developed, slides were removed and the reaction was stopped by immersion in PBS. Negative controls were carried out with rabbit serum diluted to the same concentration as the primary antibody. MBOAT7 immunostaining was evaluated in a blinded fashion regarding any of the histological and clinical characteristics of the patients. The extent of staining was scored according to its amount and intensity by a 4-point scoring system as follows: 0 = no staining, 1 = positive staining in less than 20% of cells, 2 = 21–50% of positive cells, and 3 = positive staining in more than 50% of cells. The sections were observed in bright field microscopy with a microscope Axiostar plus (Carl Zeiss, Germany) at a magnification of X400. As control tissue we used a sample of testis retrieved from the collection of tissues of the Pathology Department.

### Statistical analysis

Quantitative data were expressed as mean ± SD unless otherwise indicated. As a significant difference in SD was observed between the groups in most of the variables and the distribution was significantly skewed in most cases, we chose to be conservative and assessed the differences between the groups using nonparametric Mann–Whitney *U* or Kruskal-Wallis tests. The Cochran–Armitage test for trend was used in the categorical data analysis to assess the presence of association between the variant and disease severity and a regression analysis for an ordinal multinomial distribution (Probit as the Link function) with disease severity as the dependent (response) variable coding controls; NAFL and NASH subjects as 0, 1, and 2, respectively; age, HOMA, and BMI as continuous predictor variables; and sex and rs641738 genotypes (0, 1, 2) as grouping variables. Moreover, logistic regression analysis was included for the evaluation of the association between genotypes and histological disease severity (NAS, ballooning, fibrosis, and inflammation: present coded as 1 or absent coded as 0). To assess the association between genotypes with NAFLD or quantitative traits, we used a chi-square test and logistic regression or ANCOVA and multiple regression, adjusting for co-variables, such as age, HOMA, BMI, and rs738409. For ordinal multinomial analysis, logistic analysis, or ANCOVA, we adjusted for co-variables that were not normally distributed through log-transformation. Correlation between two variables was done using the Spearman’s rank correlation test. The CSS/Statistica program package version 6.0 (StatSoft, Tulsa, OK, USA) was used in these analyses.

### Data availability

All data generated or analyzed during this study are included in this published article.
